# Anterior mediastinal epithelioid hemangioendothelioma associated with myelolipoma and extramedullary hematopoiesis mimicking a mature teratoma: A radiologic-pathologic correlation

**DOI:** 10.1016/j.radcr.2026.09.030

**Published:** 2026-10-12

**Authors:** Saubhagya Dhakal, Paulo Savoia, Subhashis Mitra, Manisha Sharma

**Affiliations:** a Department of Radiology, University of Iowa Hospitals & Clinics, Iowa City, IA, USA; b Department of Pathology, University of Iowa Hospitals & Clinics, Iowa City, IA, USA; c Department of Pediatrics, University of Connecticut, Hartford, CT, USA

**Keywords:** Epithelioid hemangioendothelioma, Anterior mediastinum, Myelolipoma, Extramedullary hematopoiesis, Mature teratoma

## Abstract

Epithelioid hemangioendothelioma (EHE) is an ultra-rare vascular neoplasm with variable biologic behavior. Mediastinal EHE is particularly uncommon and may mimic more prevalent prevascular mediastinal tumors. We report a 52-year-old man with a 10.5 × 8.9 × 9.6 cm mixed-density left upper anterior mediastinal mass containing macroscopic fat, soft-tissue components, and coarse calcification. FDG PET/CT demonstrated mild-to-moderate uptake (SUVmax 4.8) without nodal or distant metastatic disease, and the imaging appearance favored mature teratoma. Resection demonstrated a 9.5 cm conventional EHE intimately associated with a myelolipomatous component containing extensive extramedullary hematopoiesis and metaplastic ossification. The neoplastic cells expressed endothelial markers, and RNA fusion analysis detected WWTR1::CAMTA1. Surgical margins were negative, early postoperative and abdominopelvic imaging showed no residual or metastatic disease, and multidisciplinary review recommended surveillance without adjuvant therapy. Although similar EHE–myelolipoma/extramedullary hematopoiesis associations have been reported, this case emphasizes the multimodality imaging mimic of mature teratoma and provides direct radiologic-pathologic correlation of the fat-containing, solid, and ossified components.

## Introduction

Epithelioid hemangioendothelioma (EHE) is a rare vascular neoplasm characterized by an intermediate-to-malignant biologic potential and an unpredictable clinical course. Conventional EHE typically demonstrates epithelioid endothelial cells arranged in cords, strands, or small nests within a myxohyaline stroma. Most conventional tumors harbor a WWTR1::CAMTA1 fusion, whereas a smaller subset is characterized by YAP1::TFE3 rearrangement [1].

Mediastinal EHE is particularly rare. Its imaging features may overlap with more common prevascular mediastinal lesions, including thymic epithelial neoplasms, germ-cell tumors, lymphoma, lipomatous tumors, and myelolipoma. Fat and coarse calcification within an anterior mediastinal mass strongly suggest mature teratoma, although uncommon composite or mixed neoplasms can have a similar appearance [[2], [3], [4], [5]].

We report a large anterior mediastinal EHE intimately associated with a myelolipomatous component containing extensive extramedullary hematopoiesis (EMH) and metaplastic ossification. In this manuscript, “myelolipoma” refers to the fat-containing lesion composed of mature adipose tissue admixed with hematopoietic elements, whereas “EMH” refers specifically to the hematopoietic component; the terms are therefore related but not used as synonyms. Although similar EHE-myeloid/adipose composite lesions have been reported, the present case is distinct in its strong prospective mimicry of mature teratoma on multimodality imaging and in the component-by-component radiologic-pathologic correlation of macroscopic fat, nonfat soft tissue, and mineralization with the resection findings. The lesion was initially interpreted radiologically as a mature teratoma because of its mixed fat, soft-tissue, and calcific components.

### Case presentation

A 52-year-old man initially presented with concern for possible pneumonia. Chest radiography obtained during evaluation demonstrated a large left superior mediastinal mass. He was subsequently referred to pulmonology for further assessment.

Over the preceding several weeks, the patient reported progressive shortness of breath, dry cough, and vague dull left-sided chest pain. He had lost approximately 45 pounds after beginning work as a mail carrier and increasing his exercise level. Because weight loss of this magnitude can be a potential red flag for malignancy when unexplained, it was important to clarify the history; in this patient, the weight loss was reported as intentional and temporally related to increased physical activity rather than as an unexplained constitutional symptom. He denied any history of tobacco use and reported consuming approximately two to three alcoholic drinks nightly. No other significant symptoms were reported.

Contrast-enhanced CT of the chest demonstrated a large, well-circumscribed mixed-density mass centered in the left upper prevascular mediastinum, measuring 10.5 × 8.9 × 9.6 cm (Figs. 1B and C). The mass contained macroscopic fat, nonfat soft-tissue components, and multiple foci of coarse calcification. No mediastinal lymphadenopathy, definite vascular encasement, or other radiographic evidence of invasive growth was identified. The combination of macroscopic fat, soft tissue, and calcification strongly favored a germ-cell tumor, particularly mature teratoma; thymoma and a lipomatous neoplasm were considered less likely. In retrospect, the lesion lacked a specific imaging feature that would have confidently distinguished EHE from mature teratoma before tissue diagnosis. Its sizable nonfat solid component and mild FDG uptake were compatible with a neoplasm but were not specific for EHE.

**Fig. 1:**
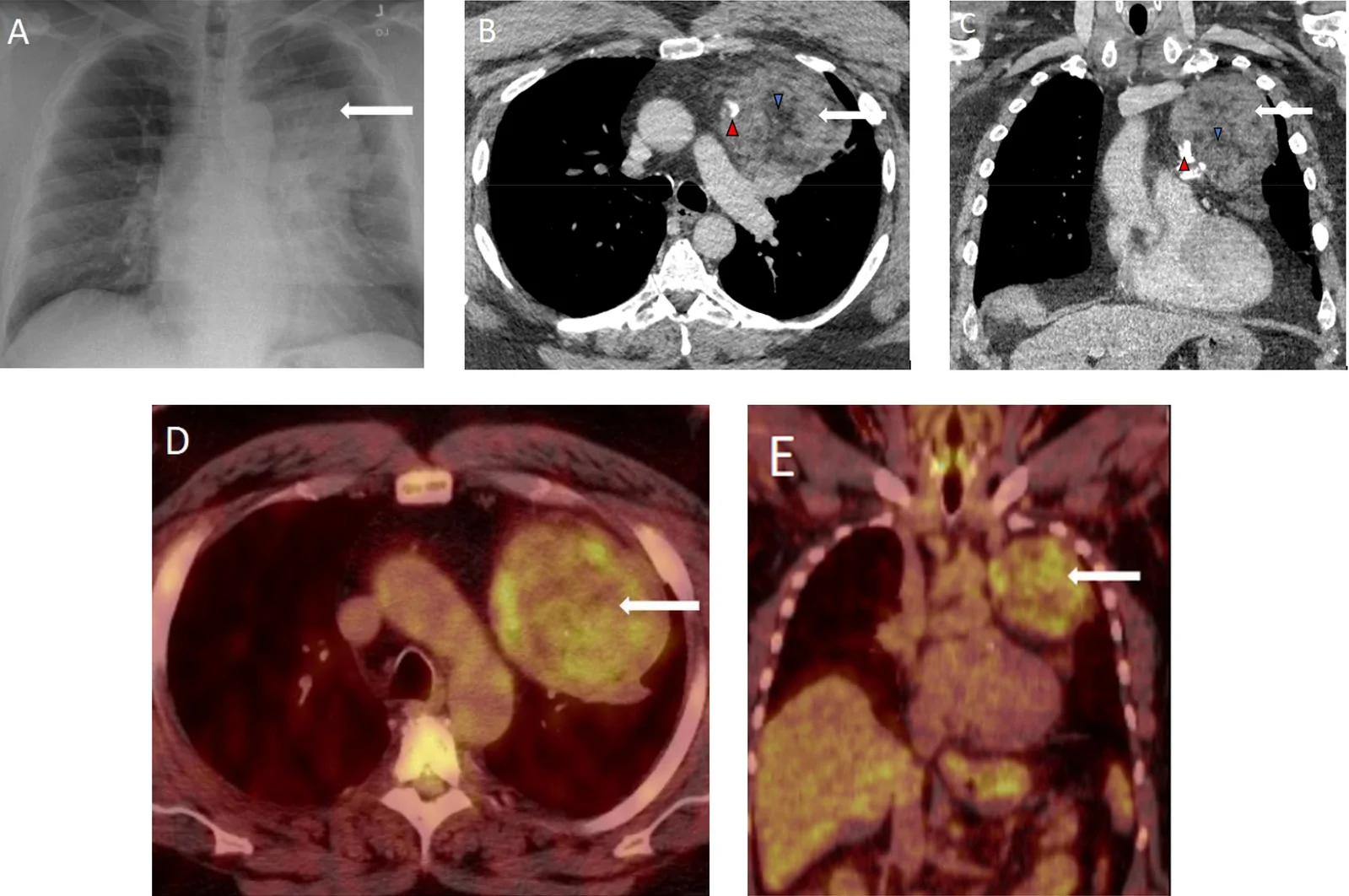
Multimodality imaging of anterior mediastinal epithelioid hemangioendothelioma. (A) Frontal chest radiograph demonstrates a well-defined left upper mediastinal opacity (white arrow). (B) Axial and (C) coronal contrast-enhanced CT images demonstrate a large, well-circumscribed mixed-density mass in the left upper prevascular mediastinum measuring 10.5 × 8.9 × 9.6 cm. The white arrows indicate the nonfat soft-tissue component, the blue arrowheads indicate macroscopic fat, and the red arrowheads indicate coarse calcification/mineralization. On radiologic-pathologic correlation, the macroscopic fat was attributable to the myelolipomatous component; the nonfat soft-tissue portions reflected intermixed EHE and hematopoietic tissue and could not be reliably separated on CT; and the mineralized foci corresponded to metaplastic ossification. (D) Axial and (E) coronal fused FDG PET/CT images show mild-to-moderate FDG uptake within the mass (white arrows), with SUVmax 4.8. No additional FDG-avid lesion was identified. The imaging appearance initially favored a mature teratoma/germ-cell tumor.

FDG PET/CT redemonstrated the left upper anterior mediastinal mass with mild-to-moderate metabolic activity (SUVmax 4.8) (Fig. 1D and E). No abnormal FDG-avid lymphadenopathy or distant hypermetabolic lesion was identified. This degree of uptake is within the broad range reported for EHE and therefore does not exclude the diagnosis; however, FDG avidity is variable and is not sufficiently specific to distinguish EHE from other anterior mediastinal masses. In this setting, PET/CT was most useful for whole-body staging rather than for establishing the histologic diagnosis [6]. Serum alpha-fetoprotein, beta-human chorionic gonadotropin, and lactate dehydrogenase levels were not elevated.

The patient underwent left hemiclamshell thoracotomy with anterior mediastinal mass resection, partial thymectomy, and intercostal cryoablation at the fourth, fifth, and sixth intercostal spaces. The mass was densely adherent to the phrenic nerve and innominate vein intraoperatively, but no gross invasion was documented. The pathology report did not document microscopic invasion of these structures, and all surgical margins were negative. Thus, the operative adherence was interpreted as dense adhesion rather than proven macroscopic or microscopic invasion.

Postoperative contrast-enhanced CT of the chest showed expected postsurgical changes without a residual mediastinal mass, together with a moderate left pleural effusion and adjacent compressive atelectasis. Ultrasound-guided thoracentesis removed approximately 1350 mL of serosanguinous/dark bloody fluid; pleural-fluid cytology was negative for tumor cells. CT of the abdomen and pelvis showed no evidence of metastatic disease. At oncology follow-up, the patient was recovering well and denied chest pain, shortness of breath, abdominal pain, constitutional symptoms, or other systemic complaints. Following multidisciplinary tumor-board review, no additional therapy was recommended after complete resection with negative margins. The surveillance plan was cross-sectional imaging at approximately 6 months after resection and annually thereafter.

### Pathologic findings

Surgical pathology demonstrated a 9.5 cm anterior mediastinal mass containing a myelolipomatous component composed of mature adipose tissue admixed with extensive extramedullary hematopoiesis, together with foci of solid tumor with indistinct borders (Figs. 2A–C). This terminology is used throughout the revised manuscript to distinguish the myelolipoma as the fat-containing lesion from EMH as its hematopoietic component rather than to imply two separate, unrelated lesions.

**Fig. 2:**
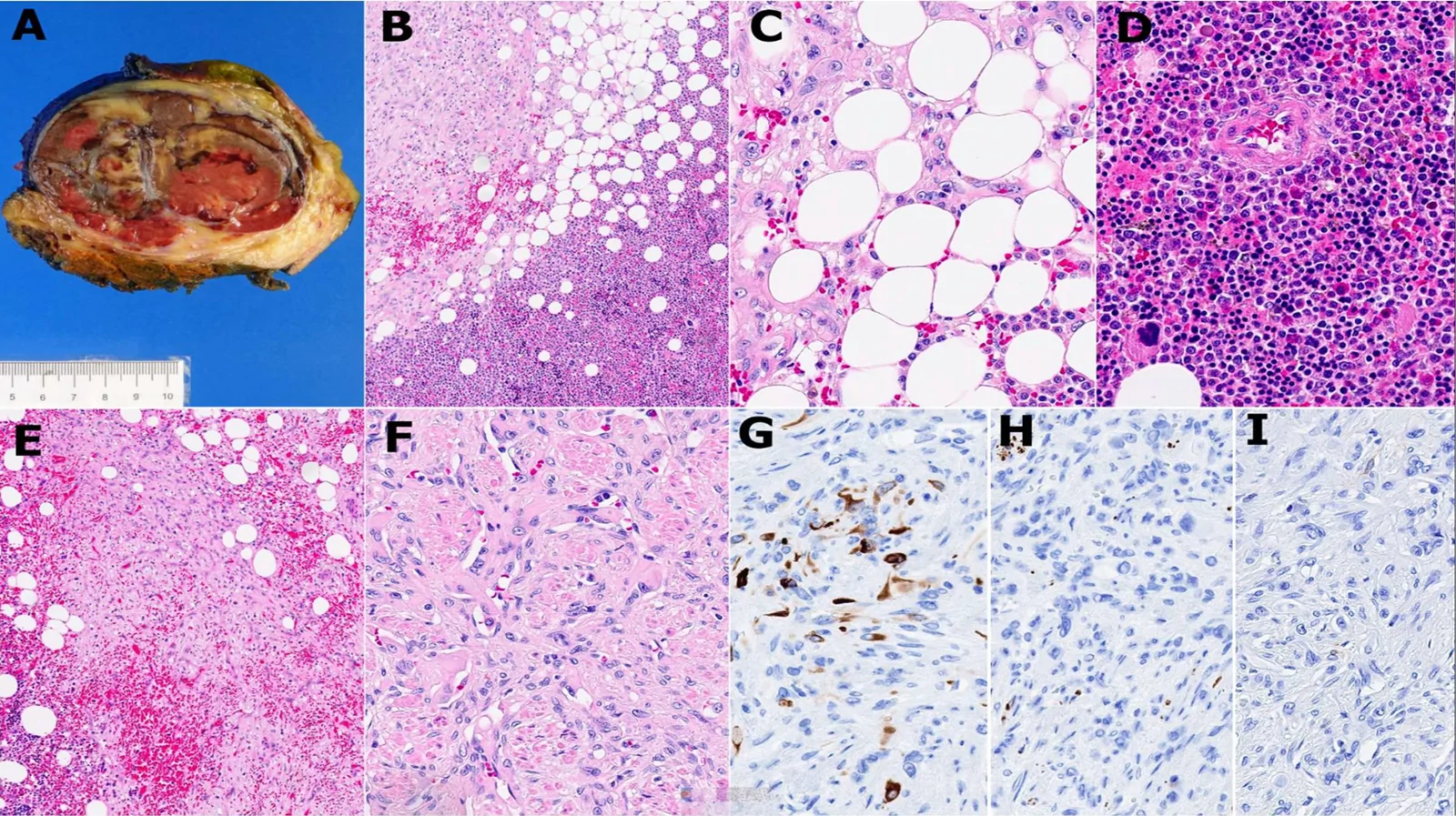
Gross and histopathologic features of anterior mediastinal epithelioid hemangioendothelioma associated with a myelolipomatous component containing extramedullary hematopoiesis. (A) Macroscopic view of the resected tumor. (B) Hematoxylin and eosin (H&E), × 10, low-power view showing the myelolipomatous component with mature adipose tissue and hematopoietic elements. (C) H&E, × 40, high-power view demonstrating hematopoietic elements adjacent to solid EHE. (D) H&E, × 10, low-power view highlighting extensive extramedullary hematopoiesis. (E) H&E, × 10, low-power view of solid EHE in a myxoid background. (F) H&E, × 40, high-power view demonstrating epithelioid and spindled tumor cells arranged in cords, singly, and in small aggregates, with occasional intracytoplasmic vacuoles. Immunohistochemistry demonstrates neoplastic cells that are (G) focally positive for pancytokeratin and negative for (H) desmin and (I) S100.

Microscopically, the myelolipomatous component showed abundant mature adipose tissue and extensive hematopoietic elements (Figs. 2B–D). Intimately associated solid foci were composed of epithelioid-to-spindled neoplastic cells arranged in cords, singly, and in small aggregates within a somewhat myxoid matrix (Figs. 2E and F). Occasional intracytoplasmic vacuoles, corresponding to "blister cells," were present. Mild nuclear pleomorphism and metaplastic ossification were also identified. Radiologic-pathologic correlation explained the heterogeneous CT appearance: the macroscopic fat was attributable to the myelolipomatous component, whereas the nonfat soft-tissue portions reflected intermixed EHE and hematopoietic tissue and could not be reliably separated from one another on CT; the mineralized foci corresponded to metaplastic ossification.

The mitotic rate was low, approximately 1 mitosis/mm², and no significant necrosis was identified. Immunohistochemistry showed focal pancytokeratin positivity, with negative desmin and S100 staining (Figs. 2G–I). The neoplastic cells were positive for smooth muscle actin (SMA), CD31, CD34, and ERG and negative for TFE3, STAT6, HHV8, and HMB45 (Figs. 3A–H). Additional negative stains included Melan-A. This immunophenotype supported epithelioid hemangioendothelioma.

**Fig. 3:**
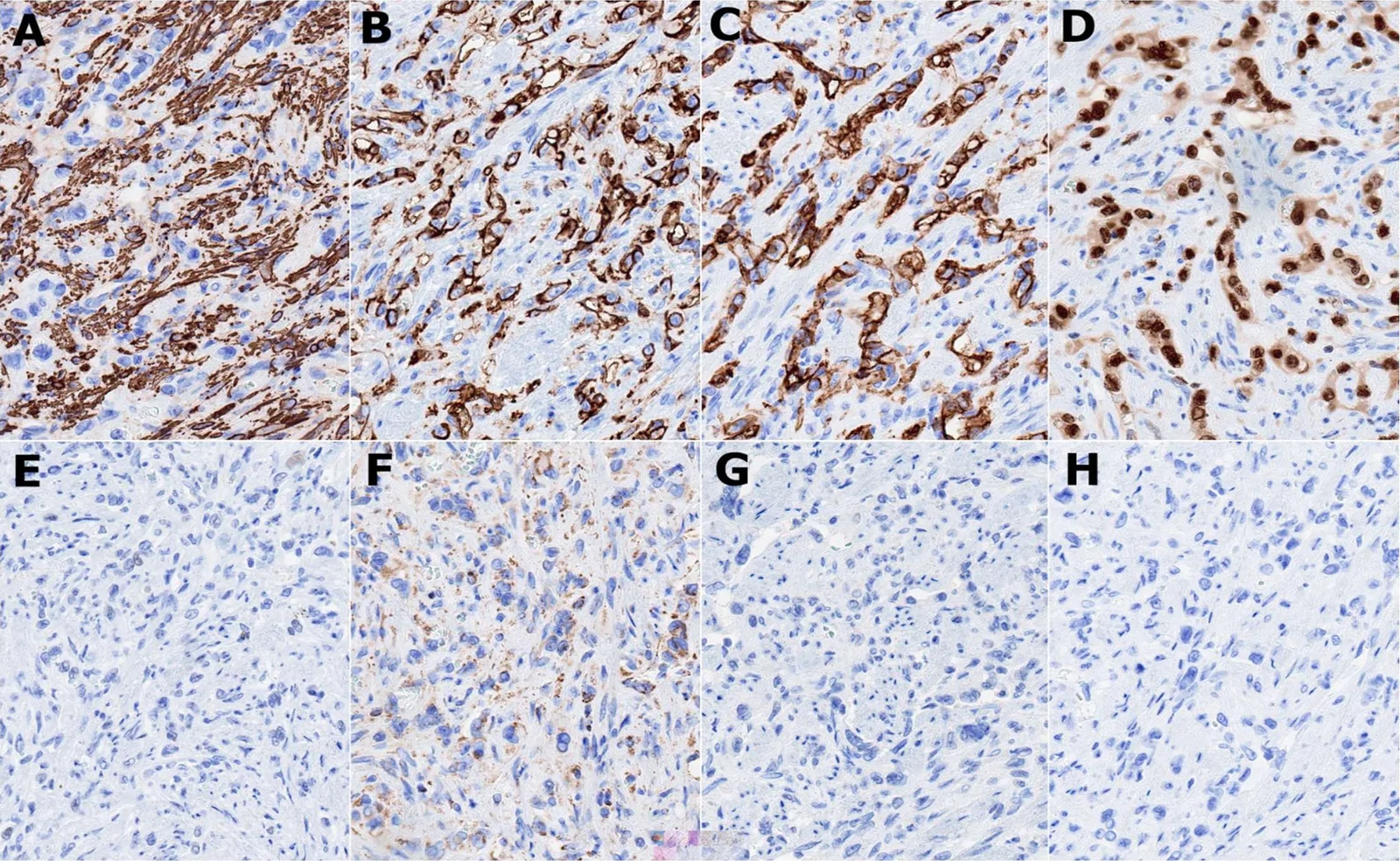
Immunohistochemical profile of anterior mediastinal epithelioid hemangioendothelioma. Immunohistochemistry demonstrates neoplastic cells positive for (A) smooth muscle actin (SMA), (B) CD31, (C) CD34, and (D) ERG and negative for (E) TFE3, (F) STAT6, (G) HHV8, and (H) HMB45, consistent with epithelioid hemangioendothelioma.

Fluorescence in situ hybridization was negative for MDM2 amplification, excluding dedifferentiated liposarcoma, an important diagnostic consideration in a large fat-containing mediastinal lesion. Targeted next-generation DNA sequencing did not identify reportable pathogenic mutations. RNA fusion analysis detected a WWTR1 exon 3::CAMTA1 exon 8 fusion transcript, consistent with the recurrent t(1;3)(p36;q25) rearrangement characteristic of conventional EHE. All surgical margins were negative.

Taken together, the morphologic, immunophenotypic, and molecular findings confirmed conventional epithelioid hemangioendothelioma intimately associated with a myelolipomatous component containing extensive extramedullary hematopoiesis and metaplastic ossification.

## Discussion

EHE is an ultra-rare endothelial neoplasm with variable biologic behavior. Conventional EHE is characterized histologically by epithelioid endothelial cells in cords, strands, or small aggregates within a myxohyaline stroma, often with intracytoplasmic vacuoles. Endothelial differentiation is supported by CD31, ERG, and CD34 expression, while focal cytokeratin expression may create diagnostic confusion with carcinoma or thymic epithelial tumors. Most conventional EHEs harbor WWTR1::CAMTA1 fusion [1].

Mediastinal EHE is uncommon and can demonstrate local invasion or metastatic disease [7]. In the present case, the characteristic morphology, endothelial immunophenotype, and WWTR1::CAMTA1 fusion established the diagnosis despite an imaging appearance that strongly suggested a germ-cell tumor.

The most important radiologic feature of this case was the combination of macroscopic fat, nonfat soft tissue, and coarse mineralization in a prevascular mediastinal mass. This pattern is highly characteristic of mature teratoma, in which fat and calcification are common [3]. Retrospectively, no single CT or PET feature was specific enough to favor EHE preoperatively. Rather, the case demonstrates a diagnostic pitfall: a vascular neoplasm can acquire a teratoma-like appearance when EHE is intimately associated with a myelolipomatous/hematopoietic component and metaplastic ossification.

The principal competing diagnoses for a fat-containing anterior mediastinal mass include mature teratoma, thymolipoma, liposarcoma, and, less commonly, myelolipoma or mass-forming EMH [4,5,8,9]. In this patient, mature teratoma was favored because of the admixture of fat, soft tissue, and coarse calcification. Dedifferentiated liposarcoma remained a pathologic consideration because of the large fatty mass with solid areas, but absence of MDM2 amplification argued against that diagnosis.

For clarity, myelolipoma and EMH are related but not identical terms. Myelolipoma is a mass-forming lesion composed of mature adipose tissue admixed with hematopoietic elements, whereas EMH describes hematopoiesis occurring outside the normal marrow compartment. In the present lesion, the pathology showed a myelolipomatous component with extensive EMH rather than two spatially independent masses. This distinction also helps explain the imaging appearance: the mature adipose tissue produced macroscopic fat attenuation, while the hematopoietic and neoplastic components contributed nonfat soft-tissue attenuation.

The association of EHE with myelolipomatous/hematopoietic tissue has been reported previously, so the novelty of the present case is not the first description of this combination. Diaz-Perez et al. described conventional EHE arising within a mediastinal myelolipoma and discussed a possible WWTR1-related basis for a true composite neoplasm [10]. This is biologically intriguing because WWTR1 alterations have been implicated in conventional EHE and have also been reported in myelolipoma, raising the possibility of a shared molecular susceptibility; however, a causal relationship has not been established. Patrini et al. described mediastinal EHE with fat, metaplastic bone, and extramedullary hematopoiesis [11]. Pinczewski and Papadimitriou proposed a different, non-mutually exclusive mechanism in which aberrant VEGF signaling by EHE could recruit or expand extramedullary hematopoietic tissue [12]. These mechanisms remain hypotheses rather than proven explanations. The present case adds a particularly strong radiologic-pathologic teaching correlation: a large prevascular lesion with macroscopic fat, solid tissue, coarse mineralization, and only moderate FDG uptake was prospectively interpreted as mature teratoma, while resection demonstrated that these imaging components corresponded to myelolipomatous tissue/EMH, intermixed EHE and hematopoietic tissue, and metaplastic ossification. Molecular confirmation of WWTR1::CAMTA1 establishes conventional EHE and strengthens the interpretation of a genuine composite association rather than an incidental imaging overlap.

The operative findings also illustrate the limits of imaging for assessing local involvement. Preoperative CT showed no definite vascular encasement or gross invasive growth, yet the mass was densely adherent to the phrenic nerve and innominate vein at surgery. No gross invasion was documented, no microscopic invasion of these structures was reported pathologically, and the resection margins were negative. Accordingly, dense operative adherence in this case should not be equated with proven invasion.

Complete surgical excision with negative margins is the preferred approach for localized, resectable EHE [1]. Because EHE can recur or progress after apparently complete resection, continued imaging surveillance is appropriate. Expert consensus supports CT-based surveillance, with follow-up imaging tailored to sites identified at baseline [1]. In this patient, early postoperative chest CT showed no residual mediastinal mass, pleural-fluid cytology after thoracentesis was negative for tumor cells, and CT of the abdomen and pelvis showed no metastatic disease. Multidisciplinary tumor-board review recommended no adjuvant therapy after negative-margin resection and planned surveillance imaging at 6 months and then annually. Although these findings provide additional early postoperative and metastatic assessment, the duration of follow-up remains short and does not permit assessment of delayed local recurrence or metastatic progression; this remains an important limitation of the case report.

## Conclusion

Epithelioid hemangioendothelioma should be considered as a rare mimic of a fat-containing and calcified prevascular mediastinal mass when pathology demonstrates a vascular neoplasm intimately associated with myelolipomatous/hematopoietic tissue. In this case, imaging was essentially indistinguishable from mature teratoma before resection. Direct radiologic-pathologic correlation showed that the macroscopic fat, nonfat solid tissue, and mineralized foci reflected the myelolipomatous component with EMH, EHE, and metaplastic ossification, respectively. Endothelial immunophenotyping and WWTR1::CAMTA1 fusion confirmed conventional EHE.

## Teaching point

A fat-containing and calcified anterior mediastinal mass can closely mimic mature teratoma yet rarely represent conventional EHE intimately associated with a myelolipomatous component containing extramedullary hematopoiesis and metaplastic ossification; correlation of the imaging components with pathology is essential for understanding this diagnostic pitfall.

## Ethics statement

This report describes a single de-identified patient case. Institutional review board approval was not required for publication of this case report in accordance with institutional policy.

## Author contributions

Saubhagya Dhakal: Conceptualization, imaging review, literature review, manuscript drafting, and manuscript revision. Paulo Savoia: Imaging review, case interpretation, and manuscript revision. Subhashis Mitra: Pathologic review, molecular-pathology interpretation, and manuscript revision. Manisha Sharma: Clinical review, literature review, and manuscript revision. All authors reviewed and approved the final manuscript.

## Patient consent

Written informed consent for publication of this case report and accompanying de-identified images was obtained from the patient.
